# Predicting Adherence to Internet-Delivered Psychotherapy for Symptoms of Depression and Anxiety After Myocardial Infarction: Machine Learning Insights From the U-CARE Heart Randomized Controlled Trial

**DOI:** 10.2196/10754

**Published:** 2018-10-10

**Authors:** John Wallert, Emelie Gustafson, Claes Held, Guy Madison, Fredrika Norlund, Louise von Essen, Erik Martin Gustaf Olsson

**Affiliations:** 1 Clinical Psychology in Healthcare Department of Women's and Children's Health Uppsala University Uppsala Sweden; 2 Uppsala Clinical Research Center Uppsala University Uppsala Sweden; 3 Department of Medical Sciences Uppsala University Uppsala Sweden; 4 Department of Psychology Umeå University Umeå Sweden

**Keywords:** applied predictive modeling, cardiac rehabilitation, linguistics, supervised machine learning, recursive feature elimination, treatment adherence and compliance, Web-based interventions

## Abstract

**Background:**

Low adherence to recommended treatments is a multifactorial problem for patients in rehabilitation after myocardial infarction (MI). In a nationwide trial of internet-delivered cognitive behavior therapy (iCBT) for the high-risk subgroup of patients with MI also reporting symptoms of anxiety, depression, or both (MI-ANXDEP), adherence was low. Since low adherence to psychotherapy leads to a waste of therapeutic resources and risky treatment abortion in MI-ANXDEP patients, identifying early predictors for adherence is potentially valuable for effective targeted care.

**Objectives:**

The goal of the research was to use supervised machine learning to investigate both established and novel predictors for iCBT adherence in MI-ANXDEP patients.

**Methods:**

Data were from 90 MI-ANXDEP patients recruited from 25 hospitals in Sweden and randomized to treatment in the iCBT trial Uppsala University Psychosocial Care Programme (U-CARE) Heart study. Time point of prediction was at completion of the first homework assignment. Adherence was defined as having completed more than 2 homework assignments within the 14-week treatment period. A supervised machine learning procedure was applied to identify the most potent predictors for adherence available at the first treatment session from a range of demographic, clinical, psychometric, and linguistic predictors. The internal binary classifier was a random forest model within a 3×10–fold cross-validated recursive feature elimination (RFE) resampling which selected the final predictor subset that best differentiated adherers versus nonadherers.

**Results:**

Patient mean age was 58.4 years (SD 9.4), 62% (56/90) were men, and 48% (43/90) were adherent. Out of the 34 potential predictors for adherence, RFE selected an optimal subset of 56% (19/34; Accuracy 0.64, 95% CI 0.61-0.68, *P*<.001). The strongest predictors for adherence were, in order of importance, (1) self-assessed cardiac-related fear, (2) sex, and (3) the number of words the patient used to answer the first homework assignment.

**Conclusions:**

For developing and testing effective iCBT interventions, investigating factors that predict adherence is important. Adherence to iCBT for MI-ANXDEP patients in the U-CARE Heart trial was best predicted by cardiac-related fear and sex, consistent with previous research, but also by novel linguistic predictors from written patient behavior which conceivably indicate verbal ability or therapeutic alliance. Future research should investigate potential causal mechanisms and seek to determine what underlying constructs the linguistic predictors tap into. Whether these findings replicate for other interventions outside of Sweden, in larger samples, and for patients with other conditions who are offered iCBT should also be investigated.

**Trial registration:**

ClinicalTrials.gov NCT01504191; https://clinicaltrials.gov/ct2/show/NCT01504191 (Archived at Webcite at http://www.webcitation.org/6xWWSEQ22)

## Introduction

Myocardial infarction (MI) afflicts more than 7 million individuals each year, making it the most common acute cardiac event caused by cardiovascular disease (CVD)—the leading cause of death in the world [1]. After an acute MI, behavior changes are required in order to reduce the risk of reinfarction, stroke, and death. Important health-promoting behaviors include smoking cessation, regular physical activity, a healthy diet, and adherence to medications [2,3].

A substantial subgroup of patients with MI additionally also suffer from symptoms of anxiety, depression, or both (MI-ANXDEP). MI-ANXDEP patients have a higher risk factor burden and worse prognosis compared to MI patients in general [4,5]. Alongside prescribed physical activity, psychological support is therefore suggested as treatment for MI-ANXDEP patients to reduce affective symptoms [6-8] and in turn facilitate health-promoting behavioral change toward cardiac risk reduction [2,9]. Psychological support in the form of cognitive behavior therapy (CBT) has shown effectiveness on psychological symptoms for several common psychiatric disorders. Internet-delivered CBT (iCBT) is a cost-effective version of face-to-face CBT [10,11] that, however, places high demands on the reading and writing abilities of the patient. Patient dropout from iCBT in a meta-analysis for depression (n=40 studies) was 57%. Subanalyses showed 28% and 38% dropout from therapist- and administrator-supported iCBT, respectively. These attrition numbers are substantial, suggesting further research into adherence to iCBT. Although adherence to iCBT is not a guarantor for iCBT effectiveness, adherence is arguably a prerequisite for possible iCBT effect and thus worthwhile to study in its own right [12].

The multicenter Uppsala University Psychosocial Care Programme (U-CARE) Heart study was the first randomized controlled trial to test the effectiveness of a therapist-supported iCBT treatment for MI-ANXDEP patients [13,14]. The U-CARE Heart trial design arguably had high ecological (clinical routine) validity [14] compared to other iCBT trials that have relied on self-referral and applied stricter inclusion/exclusion criteria [15,16]. U-CARE Heart also featured relatively low adherence to iCBT, which in turn lacked effect at the group comparison level [13]. For future dissemination of iCBT, it is crucial to assess the effect and practical utility of iCBT under ecologically valid conditions [17] such as in U-CARE Heart and explore factors that predict adherence if low adherence is a problem in such contexts. Adherence to treatment by cardiovascular patients has been thoroughly investigated with respect to medical compliance [18] but not with respect to iCBT offered to MI-ANXDEP patients.

Treatment adherence is in general a multifactorial phenomenon. Adherence to and effectiveness of iCBT has been associated with higher education, older age, and female sex [19,20]. In addition to these background predictors, both patient motivation [12] and treatment credibility [19] have been found to substantially augment adherence to iCBT. Regarding MI-ANXDEP psychological symptomatology, patient motivation and belief in the iCBT treatment are probably also predicated on cardiac-related anxious and depressive symptom severity as well as placebo priors regarding iCBT effectiveness. The so-called therapeutic alliance, the patient-therapist bond sought to be developed during individual psychotherapy, has also been found to benefit adherence to iCBT [12]. Furthermore, it is worthwhile to investigate the relative predictive power of some cardiovascular variables, as somatic disease severity might also influence adherence to iCBT among MI-ANXDEP patients.

The present iCBT U-CARE Heart study design offered a group of additional predictors that have not been assessed in this way, namely linguistic variables based on the texts that patients wrote in response to their standardized homework assignments. Syntactic structure and word use has to some extent been investigated before with regard to anxiety and depression [21-23], and number of words used when applying for Web-based depression treatment has been shown to correlate with adherence [24]. In the U-CARE Heart study, the texts are logged at the start of treatment, and various quantitative variables can be extracted from these texts using linguistic procedures. These extracts were then modeled as additional linguistic predictors for adherence in our study. It is likely that more verbally oriented and engaged patients write longer and more complex texts and also adhere better to verbally demanding treatments such as iCBT. It is also possible that these linguistic predictors to some extent are proxies of other established predictors for adherence (eg, motivation, treatment credibility, and therapeutic alliance) and as proxies would thus hold predictive power. We propose that these linguistic predictors might contribute to the acuity of predictive models in addition to known predictors of iCBT adherence (eg, education, age, sex, and symptom severity).

The objective of our study was to investigate if predictors available up to the start of treatment (initial homework assignment response) would predict adherence to iCBT treatment at first follow-up in MI-ANXDEP patients. To this end, we applied a contemporary machine learning procedure to U-CARE Heart data to manage the relatively large amount of predictors and complex covariance structure. We hypothesized that symptom severity, age, sex, education, and linguistic behavior would predict adherence to treatment. We also hypothesized that more severe symptoms, younger age, being a woman, having a higher education, and using more words in the assignment response would be positively associated with adherence to iCBT.

## Methods

### Treatment and Study Sample

The recruitment, treatment, and follow-up of patients has been described in detail elsewhere [13,14,25]. In summary, the trial recruited 239 patients from 25 Swedish hospitals and randomized 122 patients to a control group and 117 patients to therapist-guided and self-tailored 14-weeks of iCBT. Of these 117 patients, 27 did not respond to any homework assignments and were excluded due to lack of data on all linguistic variables. This rendered a study sample of 90 patients. The treatment modules consist of homework assignments to be completed by the patient on which the licensed psychologist provided feedback. The psychologist communicated with the patient through an in-portal message system. The first two homework assignments were standardized for all patients. This standardization removed the problem of complex patient-psychologist interactions that are inherently dynamic. After the first two assignments, the treatment was self-tailored. The treatment consisted of psychoeducation on principals for rational versus irrational thinking, graded exposure to fearful stimuli, the negative feedback loop in depressive behavior, as well as relaxation training, improving communication skills, additional behavioral change toward long-term goals, and relapse prevention.

### Outcome and Initial Predictor Selection

The outcome variable was dichotomous: adherence was defined as completing 3 or more homework assignments (≥21% of total treatment), and nonadherence was defined as having completed less than that. This cutoff was chosen in part because it is clinically relevant to ascertain who continues with the self-tailored part of the U-CARE Heart treatment after completing the initial 2 standardized homework assignments versus who does not continue. Furthermore, the chosen cutoff rendered fairly balanced classes for the machine learning procedure, which is important for it to work properly with moderately sized data [26]. Psychological (EO, JW, FN), cardiologic (CH), and linguistic (EG) experts selected an initial set of 34 possible predictors of psychometric, linguistic, clinical, and demographic type. See Table 1 for further details on the predictors.

### Linguistic Predictors

The linguistic predictors were extracted from the patients’ answers to the first standardized homework assignment, which consisted of an introductory text and 8 questions designed for the patient to describe their MI, associated psychological reaction, present psychological state, present social support, and what the patient wanted from iCBT treatment. In effect, patients had access to the same material prior to carrying out their homework assignment [13,14]. Since the patients had read both example answers and an introductory text before writing their response, it is possible that the patients’ choice of words would be substantially, but also equally, primed when answering the questions. The linguistic factors investigated were (1) the number of words used, (2) average sentence length, (3) normalized frequencies (results given as n/1000 words) of adjectives or adverbs, (4) normalized frequencies of possessive pronouns, (5) normalized frequencies of personal pronouns, (6) whether or not the patient mentions the MI, and the (7) frequency of mutual usage of a small set of prespecified key words (used both in a standardized question and in a patient answer). Predictors 1 through 7 were selected on the basis of them being possibly indicative of adherence to iCBT as probable proxies for verbal skill, socioeconomic status, and investment in therapy, all arguably important factors for iCBT adherence. See Multimedia Appendix 1 for further details on the linguistic predictors.

### Imputation

Five of the 34 predictors had missing data, in the order of proportion missing: number of standard glasses of alcohol consumed per week, 11% (10/90); BMI, 10% (9/90); heart rate, 7% (6/90); systolic blood pressure (SBP), 7% (6/90); and the number of days between hospital admission for MI and study randomization, 4% (4/90). Missing values were thus relatively few and not considered missing completely at random (MCAR), instead their missingness was assumedly related to the other measured variables (MAR). We also did not impute the outcome. Thus, *k* nearest neighbor (*k*-NN) imputation was performed with number of nearest cases (*k*) set to 3 and all variables with missing values imputing the median of *k* values. The *k*-NN is a well-established algorithm for imputing both numerical and categorical variables based on a generalized distance metric [33,34]. In this study, the Hower distance metric was used [35]. If *k*, from which the algorithm borrows values for cases with missing values, is set low (eg, *k* ≤3), imputation with *k*-NN also preserves much of the underlying correlational structure of data.

### Predictive Modeling

Adherence is a multifactorial problem [18,20], which suggested a multivariable prediction model. For testing the relative power of predictors, a useful method would be one that can weigh the variables according to their relative importance for solving the binary classification problem of predicting adherence versus nonadherence. The Breiman random forest model [36,37] is a well-established ensemble method which usually performs well with moderately sized data, is insensitive to multicollinearity and nuisance variables, and has previously worked well with MI patient data [38]. These model characteristics are suitable for the multiple highly correlated psychometric measures and 90 MI-ANXDEP patients in this study. Random forest also models linear and higher-order effects automatically, which concurs with the main study objective to estimate the total relative importance of a range of predictors.

**Table 1:** Descriptive statistics for all treated patients with myocardial infarction and stratified by adherence to internet-delivered cognitive behavioral therapy.

Variables				All (n=90)	Adherers (n=43)	Nonadherers (n=47)	*P* value	Missing
**Demographics**								
	Age (years) mean (SD)			58.4 (9.4)	57.0 (10.4)	60.0 (8.3)	.17	0
	Women, n (%)			34 (38)	23 (54)	11 (23)	.006	0
	**Civic status, n (%)**						.80	0
		Single		15 (17)	8 (19)	7 (15)		
		Cohabitant/married		72 (80)	34 (79)	38 (81)		
		Not single but living alone		3 (3)	1 (2)	2 (4)		
	**Education (highest attained) n (%)**						.79	0
		Elementary		14 (16)	5 (12)	9 (19)		
		High school		31 (34)	16 (37)	15 (32)		
		University ≤3 years		20 (22)	10 (23)	10 (21)		
		University >3 years		25 (28)	12 (28)	13 (28)		
	Country of birth, n (%)			17 (19)	8 (19)	9 (19)	>.99	0
**Clinical**								
	Heart rate, mean (SD)			77.0 (20.4)	77.6 (21.3)	76.5 (19.7)	.81	6
	SBP^a^, mean (SD)			149.5 (32.0)	150.5 (28.2)	148.5 (35.6)	.78	6
	BMI^b^, mean (SD)			27.9 (5.0)	27.9 (5.8)	28.0 (4.3)	.89	9
	Alcohol (glasses/week), median (IQR^c^)			2.0 (0.0, 7.3)	2.0 (0.0, 8.5)	2.0 (0.0, 5.0)	.44	10
	Current smoker, n (%)			4 (4)	2 (5)	2 (4)	>.99	0
	CVD^d^ medication adherence, n (%)			18 (20)	11 (26)	7 (15)	.32	0
	**Psychoactive medication, n (%)**						.45	0
		None		75 (83)	34 (79)	41 (88)		
		As needed		6 (7)	3 (7)	3 (6)		
		Regularly		7 (8)	4 (9)	3 (6)		
		Regularly and as needed		2 (2)	2 (5)	0 (0)		
	**Other current counseling, n (%)**						.48	0
		No		67 (74)	31 (72)	36 (77)		
		≥Once per year, <once per month		9 (10)	6 (14)	3 (6)		
		≥Once per month		14 (16)	6 (14)	8 (17)		
**Psychometric, mean (SD)**								
	CAQ^e^ fear			12.7 (6.0)	14.6 (5.4)	11.0 (6.0)	.004	0
	CAQ avoidance			7.3 (4.4)	7.4 (4.2)	7.1 (4.7)	.74	0
	CAQ attention			5.7 (3.2)	6.4 (3.4)	5.1 (3.0)	.05	0
	CAQ total			25.7 (10.0)	28.4 (9.8)	23.2 (9.6)	.01	0
	ESSI^f^ total			20.1 (4.4)	20.4 (4.0)	19.7 (4.7)	.49	0
	EQ5D^g^ VAS^h^			66.0 (16.8)	64.7 (15.6)	67.2 (17.9)	.48	0
	EQ5D emotional distress			1.0 (0.5)	1.0 (0.5)	1.0 (0.4)	.84	0
	MADRS^i^ total			14.9 (6.2)	14.9 (5.7)	15.0 (6.7)	.96	0
	BADS^j^ total			21.4 (6.1)	22.4 (5.7)	20.6 (6.3)	.15	0
	HADS^k^ anxiety			10.3 (3.0)	10.5 (2.7)	10.2 (3.2)	.71	0
	HADS depression			7.9 (3.0)	8.0 (2.7)	7.9 (3.4)	.92	0
	HADS total			18.3 (4.7)	18.4 (4.0)	18.2 (5.3)	.77	0
**Linguistic**								
	Number of words, mean (SD)			306.8 (246.7)	376.8 (257.2)	242.7 (220.5)	.009	0
	Number of mutual words, mean (SD)			6.2 (5.7)	7.6 (5.9)	4.9 (5.2)	.02	0
	Sentence length, mean (SD)			13.0 (5.5)	13.6 (5.0)	12.4 (5.9)	.28	0
	Adjectives/adverbs, mean (SD)			193.2 (43.6)	187.4 (39.9)	198.5 (46.6)	.23	0
	Possessive pronouns, mean (SD)			13.1 (10.0)	12.8 (8.1)	13.4 (11.5)	.78	0
	Personal pronouns, mean (SD)			64.6 (27.1)	70.2 (24.3)	59.4 (28.8)	.06	0
	Mentions the MI^l^, n (%)			69 (77)	35 (81)	34 (72)	.44	0
**Other**								
	Days from MI to allocation, mean (SD)			70.5 (14.9)	70.3 (15.0)	70.7 (14.9)	.91	4
	**Way of preferred contact, n (%)**						.59	0
			Email	63 (70)	29 (67)	34 (72)		
			Telephone	11 (12)	5 (12)	6 (13)		
			SMS^m^	15 (17)	9 (21)	6 (13)		
			Mail	1 (1)	0 (0)	1 (2)		

^a^SBP: systolic blood pressure.

^b^BMI: body mass index.

^c^IQR: interquartile range.

^d^CVD: cardiovascular disease.

^e^CAQ: Cardiac Anxiety Questionniare [27].

^f^ESSI: ENRICHD Social Support Instrument [28].

^g^EQ5D: European Quality of Life Questionnaire–Five Dimensions.

^h^VAS: visual analog scale.

^i^MADRS: Montgomery-Asberg Depression Rating Scale [29,30].

^j^BADS: Behavioral Activation for Depression Scale–Short Form [31].

^k^HADS: Hospital Anxiety and Depression Scale [32].

^l^MI: myocardial infarction.

^m^SMS: short message service.

Although random forest already has built-in cross-validation control for overfitting through its “out-of-bag” predictions, we added a second wrapper layer around the classifier in the form of backwards algorithmic predictor selection via recursive feature elimination (RFE) resampled with 3×10–fold cross-validation [39]. This was done to further decrease the risk of overfitting and remove human bias from the final feature selection. Regular k-fold cross-validation partitions data into k parts and then trains the model k times, each time withholding data belonging to one of the folds and testing each trained model on the corresponding hold-out fold. Modeling results are thereafter usually averaged across resampling folds. Repeated cross-validation is an extension of regular k-fold cross-validation where data is again randomly partitioned into k-folds for each pass of regular cross-validation. Since random forest was used as the classifier within RFE resampling, the process optimized on classification accuracy, and predictors were ranked on their reduction in node impurity (Gini importance) across decision trees in the random forest ensemble.

### Additional Statistics

If not stated differently, we report categorical variables as count (%), numerical variables as arithmetic mean (SD), *P* value for bivariate tests of significance set at 5%, and prediction accuracy for the binary outcome (adherent vs nonadherent) with 95% confidence intervals.

### Coding

The linguistic data preprocessing was carried out with the corpus tool AntConc version 3.4.4m (Waseda University) [40], a corpus toolkit for concordancing and text analysis. Linguistic data was also annotated with a *Part of Speech-* tagger for Swedish called Stagger (Stockholm University) [41]. Analysis was done in R version 3.4.0 (The R Foundation for Statistical Computing) [42] using packages *caret, data.table, foreign, ggplot2, ggpubr, ggthemes, mice, scales, tableone,* and *VIM*.

## Results

Descriptive data are available in Table 1. Patients who were adherent to iCBT were more frequently women and had higher self-rated cardiac anxiety and cardiac anxiety specifically related to fear and attention compared to those nonadherent. Adherent patients also used more words and more mutual words in their homework assignment. There was a tendency for adherence to increase with age and higher self-rated depression. There were no significant differences between adherers and nonadherers regarding educational attainment, whether Swedish-born or not, civil status, educational attainment, clinical characteristics, days from MI to treatment allocation, or preferred way of contact.

**Figure 1:**
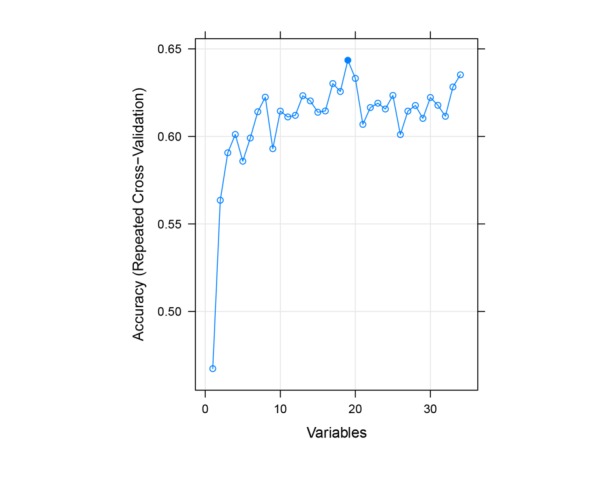
Predictor selection result with recursive feature elimination.

After imputation, the RFE feature selection procedure was applied to extract the most potent predictors for classifying adherers versus nonadherers. Figure 1 shows the resampled result optimized on prediction accuracy and the final optimal model as selected by RFE. This final model used 56% (19/34) of the provided predictors and performed significantly better than did a random model (Accuracy 0.64, 95% CI 0.61-0.68, *P*<.001) although with remaining room for acuity improvement.

**Figure 2:**
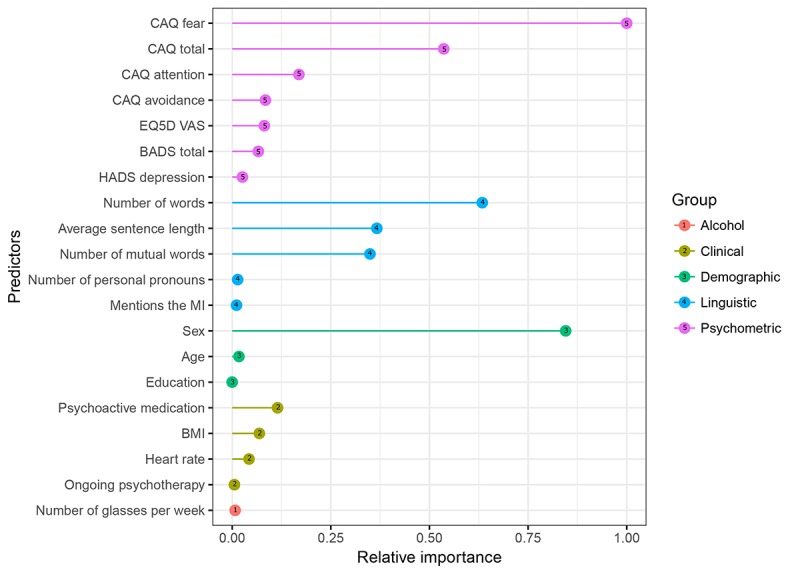
Relative importance of each predictor for adherence sorted by group. BADS: Behavioral Activation for Depression Scale–Short Form; BMI: body mass index; CAQ: Cardiac Anxiety Questionnaire; EQ5D: European Quality of Life Questionnaire–Five Dimensions; HADS: Hospital Anxiety and Depression Scale ; MI: myocardial infarction; VAS: visual analog scale.

Figure 2 plots the main result with each of the 19 top predictors according to RFE by their resampled relative importance for classifying adherers versus nonadherers, showing that the 6 most potent predictors were Cardiac Anxiety Questionnaire (CAQ) fear, sex, number of words, CAQ total, average sentence length, and number of mutual words.

## Discussion

### Principal Findings

Our study tested and compared established and novel predictors for adherence to 14 weeks of therapist-supported iCBT using data from 90 MI-ANXDEP patients recruited from 25 hospitals in Sweden and randomized to treatment in the U-CARE Heart clinical trial. The time point of prediction was after completion of the first homework assignment, which therefore allowed the study of previously untested linguistic predictors extracted from actual written behavior together with previously established predictors. A robust machine learning procedure sifted out the most potent predictors for adherence assessed at the end of treatment, which were found to be self-assessed cardiac fear, sex, number of words, self-assessed general cardiac anxiety, average sentence length, and number of mutual words used.

### Clinical Interpretation and Possible Implications

Both symptoms of general cardiac anxiety and specific cardiac fear were among the strongest predictors, and to the extent of symptom and mechanistic overlap, this corroborates previous findings that depression is associated with increased adherence to cardiac rehabilitation [43]. It is even more likely that cardiac anxiety, which is directly linked to the present treatment, would trigger activity more strongly than depression, given the respective symptomatology. Depression and anxiety are highly interconnected, which might explain the result from the cited study. Thus, patients reporting high levels of depression and patients reporting high levels of anxiety have acknowledged that they have a problem. Overall, it seems reasonable given the strength of the anxiety-based predictors that those MI-ANXDEP patients who are relatively less worried, in general and specifically regarding their heart, are less likely to adhere to treatment that specifically targets such symptoms. Our study also found that female sex was an important predictor for adherence, in line with pooled iCBT trial data confirming that males have a higher drop-out rate from Web-based interventions for depression [20]. Although not interchangeable, drop out is reasonably related to poor adherence.

On the other hand, our findings do not replicate other previously identified predictors for adherence to iCBT such as education and age [12,20], possibly due to the relatively old MI-ANXDEP patient population or the differing recruitment procedure in this study relative to the bulk of previous iCBT studies. Neither was alcohol a predictor, which might be due to a generally low level of problem drinking in the study sample. Although the U-CARE Heart inclusion had relatively high ecological validity compared to other iCBT studies, our patients were still selected, excluding, for instance, those with suicidal tendencies. Moreover, the weak predictive power of depression as gauged by the Hospital Anxiety and Depression Scale (HADS), especially compared to symptoms of anxiety and their strong predictive power, is puzzling. This may be due to exclusion of severe depressive symptoms on the basis of suicide risk, whereas no such screening was applied for very high anxious symptomatology. With that said, HADS anxiety was not a useful predictor, possibly suggesting psychometric shortcomings of the particular HADS scale. Consequently, the more cardiospecific anxiety scale CAQ seems more relevant for adherence in MI-ANXDEP patients. Furthermore, alternative ongoing treatment external to the trial (eg, psychoactive medication and third-party counseling) was not predictive of adherence to iCBT. Important to note is that there were no restrictions on patients seeking additional external treatment available from the relatively well-developed Swedish health care system. This could perhaps explain the null finding through the principle of homeostasis applied to symptom severity and sought treatment. In a relatively free and rich society, particularly severe symptomatology should be compensated for by such patients seeking and receiving multimodal treatment as needed. If so, these factors might cancel each other out with respect to both the need for and adherence to iCBT.

We also discovered that novel linguistic predictors based on written verbal responses predicted adherence. The number of words may be a proxy for verbal fluency and degree of patient effort in therapy, and the number of mutual words might be a proxy for the degree of therapeutic alliance, which in part corroborates previous research on therapeutic alliance and other interlinked concepts that promote adherence to iCBT [12,19,20,24,43]. Together with previously known predictors, these linguistic predictors may enable improved risk stratification regarding which patients will likely adhere to treatment. This suggests a largely unexplored route for future clinical research seeking to lower iCBT treatment failure and might lead to further tailoring of limited therapeutic resources for augmenting cost-effectiveness and lowering human suffering in clinical care.

Although more work is arguably needed, the data collection, preprocessing, and analysis of written responses can be automated to a considerable degree so the current lack of off-the-shelf clinical utility might not be a future obstacle. An automated tool for predicting adherence can be constructed and then possibly used as a decision support tool by the clinician. Moreover, the tool could also determine the risk of low adherence in patients, which could possibly inform the tailoring of treatment for the MI-ANXDEP patient more objectivity and accurately compared to the guesswork and crude cutoffs often applied to counter low adherence in clinical research and care today. So-called artificial intelligence and the related supervised machine learning applications that are now being rapidly researched and implemented broadly would likely also be of benefit to better solve the clinically relevant problem of predicting adherence to internet-delivered treatments.

### Limitations and Strengths

A limitation of this study is the sample size. Although the present U-CARE Heart study is the largest iCBT trial for MI-ANXDEP patients to date, it provides limited reliability estimates. The sample is too small to subdivide for more detailed analyses of those exclusively depressed or anxious. Within the limits of the present sample size not allowing for an external validation data set, the generalizability of findings is, however, quite good given that (a) applied predictive modeling procedure was robustly cross-validated, (b) national coverage was very good with recruitment from 25 hospitals, and (c) patients were recruited very similarly to routine clinical care.

Although we used expert content knowledge to select predictors and tested a range of common and domain specific predictors, there was still the possibility for using other predictors. This might explain the room for improvement in terms of classification acuity. Given that we studied a whole new class of predictors consisting of actual written behavior selected by domain experts, this study adds further novelty in that manner. The confirmation of some previously known predictors for adherence to psychotherapy with scarcely studied but very common MI-ANXDEP patients indicates potential clinical utility with MI-ANXDEP patients. The study was conducted in Sweden, and we cannot readily extrapolate our findings beyond our national and linguistic borders. The MI-ANXDEP population is also a distinct subgroup of MI patients, and the iCBT intervention is specifically tailored to these patients. Hence, replication outside of Sweden with different patients and for other psychotherapeutic treatments would be valuable.

There was also the limitation of operationalizing the outcome. This can be done in several ways, with the most strict adherence definition being to complete all treatment modules [44]. However, since the U-CARE Heart trial had particularly high ecological validity but suffered from generally low adherence [13], this cutoff definition of adherence automatically had to be low to be able to model adherence since the moderate sample size inhibited us from modeling unbalanced classes. Defining adherence as those patients continuing treatment beyond the first two standardized modules is also arguably more clinically relevant on qualitative grounds compared to an arbitrary percentage cutoff. Considering clinical needs and data availability, the patients were selected on completion of the initial standardized homework module—the optimal time to predict treatment adherence if one wants to also use linguistic predictors derived from written treatment response to make early in-treatment prediction of treatment adherence. There are also qualitative approaches to investigate adherence to iCBT [25] that might augment our understanding of adherence if combined with the current data-driven approach. Furthermore, the purpose of studying linguistic predictors automatically excluded 27 patients who were randomized to treatment but did not complete any homework assignment. For obvious reasons, our prediction model cannot generalize to these patients, yet it seems likely that prediction accuracy would theoretically be higher if including these patients because they constitute extreme cases of low adherence.

### Conclusions

For developing and testing effective iCBT interventions, investigating factors that predict adherence is important. Using a supervised machine learning approach, adherence to iCBT treatment in a multicenter trial for MI-ANXDEP patient was best predicted by a diverse set of predictors. The most potent predictors also included novel linguistic predictors from written patient behavior at the start of treatment. Our findings may improve the tailoring of iCBT for these high-risk patients. Future research should also investigate possible causal mechanisms and determine if these findings replicate outside of Sweden, in larger samples, and for other patient groups that might benefit from iCBT.

## Appendix

Multimedia Appendix 1 Supplemental material.

Multimedia Appendix 2 CONSORT-EHEALTH checklist (V 1.6.1).

## Abbreviations

CAQ: Cardiac Anxiety Questionnaire
CBT: cognitive behavioral therapy
CVD: cardiovascular disease
HADS: Hospital Anxiety and Depression Scale
iCBT: internet-based cognitive behavioral therapy
k-NN: k nearest neighbor
MI: myocardial infarction
MI-ANXDEP: myocardial infarction with comorbid symptoms of depression, anxiety, or depression and anxiety
MAR: missing at random
MCAR: missing completely at random
RFE: recursive feature elimination
SBP: systolic blood pressure
U-CARE: Uppsala University Psychosocial Care Programme
