# Supplementary material for: Predicting Adherence to Internet-Delivered Psychotherapy for Symptoms of Depression and Anxiety After Myocardial Infarction: Machine Learning Insights From the U-CARE Heart Randomized Controlled Trial
Source: J Med Internet Res. 2018 Oct 10;20(10):e10754. doi: 10.2196/10754 (PMC6234350; doi:10.2196/10754)
Supplement: Multimedia Appendix 1 [file jmir_v20i10e10754_app1.pdf]

Appendix

For

Machine Learning for Predicting Adherence to Internet-Delivered Psychotherapy for Symptoms of Depression and Anxiety after Myocardial Infarction: Insights from the U-CARE Heart Trial

By

John Wallert; Emelie Gustavson; Claes Held; Guy Madison; Fredrika Norlund; Louise von Essen; Erik Olsson

In

Journal of Medical Internet Research

A1: Linguistic keywords

The degree of mutual usage of words between the patient and the standardized questions in the first homework assignment was based on the root keywords available in Table A1. The number of times any of these words were used by a patient was counted. The search was conducted in the raw material, not the material tagged with *Parts of Speech*, and the raw value was used.

Table A1
Linguistic keywords in Swedish, English translation, and example usage.

Swedish root	English root	Examples
infark	*infarc*	Infarkt (Infarction); Hjärtinfarkt (Myocardial Infarction).
Grubb*	pond*, rum*	Grubbla (To Ponder); Grubblande (Rumination, Ruminating)
Behandl*	Treat*	Behandling (Treatment); Behandlingseffekt (Treatment effect)
Hjälp	Help	
Nedstäm*	Distress*	Nedstämnd (Distressed, singularis); Nedstämnda (Distressed, pluralis)
Oro*	Worr*(y)	Oro (Worry); Oroad (Worried)
Påverka*	Affect	Påverka (Affect); Påverkan (Affect); Påverkad (Affected)
Känsl*	Feel*	Känsla (Feeling, Emotion); Känslig (Sensitive, Fragile); Känslosam (Affective, Emotive)
Känns	Felt	
Hanter*	Handl*	Hantera (Handle), Hantering (Handling)

When the word is a root, only the root has to match in the patient’s writing for it to be counted as a mutual word.

The linguistic data processing was carried out with the corpus tool AntConc version 3.4.4m (Waseda University, Tokyo, Japan, 2016)[1], a corpus toolkit for concordancing and text analysis. Linguistic data was annotated with a *Part of Speech*-tagger for Swedish called Stagger (Stockholm University, Sweden, 2013)[2], that has a per-token accuracy about 96.6 %; the high accuracy was confirmed by manual checking of the tags for a subset of the present material.

Part of Speech-tagged data

The Part of Speech-tagged (POS-tagged) data was used to search for:
(2) average sentence length, which was achieved by searching for sentence closers *(*_MAD*) in the POS-tagged data, (with manual corrections for those patients who had left out punctuation at the end of their answers to each question) and dividing the total number of words with the adjusted number for sentence closers.

The search for: (3) normalized frequencies (results given as n/1000 words) of adjectives or adverbs (tagged with **_JJ* or **_AB*) and (4) normalized frequencies of possessive pronouns *min_PS, mitt_PS* and *mina_PS* [my, mine] was also conducted in the POS-tagged material.

In the untagged (not POS-tagged) material, the search comprised: (1) the number of words used, (5) normalized frequencies of (first person singular) personal pronouns *jag, mig, mej* [I, me] (6) whether or not the patient mentions their MI (**infark**) and (7) the key words in Table A1.

A2: Appendix references

1. *AntConc* [computer program]. Version 3.4.4. Tokyo, Japan: Waseda University; 2016.
2. Östling R. Stagger: an Open-Source Part of Speech Tagger for Swedish. *Northern European Journal of Language Technology.* 2013;3:1-18. 10.3384/nejlt.2000-1533.1331
